# Predictive Value of Serum Infliximab Levels at Induction Phase in Rheumatoid Arthritis Patients

**DOI:** 10.2174/1874312901711010075

**Published:** 2017-06-29

**Authors:** Teresa Jurado, Chamaida Plasencia-Rodríguez, Ana Martínez-Feito, Victoria Navarro-Compán, Theo Rispens, Annick de Vries, Karien Bloem, Eva-María Olariaga, Cristina Diego, Alejandro Villalba, Diana Peiteado, Laura Nuño, Maria-Gema Bonilla, Alejandro Balsa, Dora Pascual-Salcedo

**Affiliations:** 1 Immunology Unit, University Hospital La Paz, Paseo de la Castellana 261, 28046 Madrid, Spain.; 2 Rheumatology Department and Institute for Health Research (IdiPAZ), University Hospital La Paz, Madrid, Spain.; 3 Sanquin Research; Department Immunophatology; Amsterdam, The Netherland; and Landsteiner Laboratory; Academic Medical Centre; University of Amsterdam; Amsterdam, The Netherlands.

**Keywords:** Infliximab therapy, Rheumatoid Arthritis, Induction phase, Levels clearance, Loss of efficacy, Early immunogenicity

## Abstract

**Background:**

The Infliximab, has proven effective in treating rheumatoid arthritis (RA). A good clinical response is usually associated with high serum drug levels. Development of antibodies toward Infliximab (ATI) can increase drug clearance, leading to treatment failure.

**Aims:**

To analyze whether serum Infliximab trough levels (ITL) at the induction phase are associated with Infliximab clearance and clinical outcomes at week(W) 54 and to investigate the association with immunogenicity development.

**Methods:**

Observational retrospective study in which ITL from 66 RA patients were measured by capture ELISA at W0, W2, W6, W14 and 22. Patients were classified as ITLpos if Infliximab was detectable at W54 and ITLneg otherwise. ATI were assayed by bridging ELISA and by two drug-tolerant assays. ITL cut-off values were established by ROC curves. The association between ITL at early-stage and clearance of Infliximab at W54 was analyzed by univariable and multivariable logistic regression.

**Results:**

ITLneg patients (n=25) always had significantly lower Infliximab levels than ITLpos (n=41). An ITL value of 4.4 μg/mL at W6 best predicted W54 Infliximab absence. In the multivariable analysis, only ITL below the cut-off at W6 (OR: 86.6; 95%CI: 6.58-1139.99) and non-use of methotrexate (OR: 6.9; 95%CI: 1.04-45.84) remained significantly associated with W54 Infliximab absence. ATI were more frequent in patients with ITL below the cut-off at W6.

**Conclusions:**

In RA, ITL at induction phase are inversely associated with Infliximab clearance and clinical outcomes at W54. ATI was the main reason for low early ITL. A predictive value of ITL at W6 was found as a useful prognostic measure of treatment efficacy.

## BACKGROUND

Infliximab (IFX), a chimeric anti-tumor necrosis factor monoclonal antibody, is effective in the treatment of rheumatoid arthritis (RA) and other inflammatory diseases [1-4]. However, treatment fails in around 40% of patients, in many cases due to immunogenicity. An important contributor to treatment failure is the presence of antibodies towards IFX (ATI). The formation of immune complexes between antibodies and biological drugs may increase drug clearance, reduce serum levels and result in a loss of efficacy [5-8].

Several publications have reported an association between serum IFX levels and clinical response [5, 6, 9, 10]. A good clinical response is usually associated with high circulating IFX levels [11, 12]. On the other hand, low or undetectable circulating IFX levels may indicate ATI development with the consequent loss of drug efficacy.

Most of the methods available to detect ATI are hindered by the simultaneous presence of drug and antibodies in serum, being the bridging enzyme-linked immunosorbent assay (ELISA) the most susceptible one to this effect [13]. Drug interference accounts for important discrepancies between studies, so current efforts are focused on the development of assays lacking drug interference that can reveal the true prevalence of immunogenicity.

The association between early-stage IFX serum concentrations, clinical outcomes and immunogenicity has recently been studied for inflammatory diseases such as RA and ulcerative colitis (UC) [12, 14]. It is not clear why some patients show faster drug clearance from treatment commencement, being the hypothesis that circulating drug levels are affected by patient characteristics associated with baseline disease activity and an early anti-drug antibody production [15, 16].

The aim of this study is to analyze whether serum IFX trough levels (ITL) at the induction phase are associated with IFX clearance and clinical outcomes in the first year of treatment.

The implication of different factors potentially accounting for the presence of low levels at early stages of treatment is also studied.

## PATIENTS AND METHODS

### Patients and Serum Samples

This observational retrospective study included 66 patients with RA recruited from a prospective observational cohort of patients treated with biological drugs at the rheumatology department of La Paz University Hospital (Madrid). Patients fulfilled the 1987 revised criteria of the American College of Rheumatology [17], had moderate or high disease activity [18] (despite previous treatment with disease-modifying anti-rheumatic drugs), and were naïve to treatment with biologics; they were followed-up for at least one year, except when lost to follow-up for medical reasons and sera from most of the visits (at 0-2-6-14-22-54 weeks) had to be available. Patients were given intravenous infusions of 3 mg/kg of IFX at week (W) 0, 2 and 6 and every 8 weeks thereafter. Serum samples were collected at baseline and immediately before each infusion. Disease activity was assessed at baseline, W22 and W54 using the Disease Activity Score for 28 joints (DAS28) measured according to the erythrocyte sedimentation rate. Therapeutic response was evaluated using the criteria of the European League Against Rheumatism (EULAR) [19]. All patients were treated with standard dose (3mg/kg) every 8 weeks.

The study was approved by La Paz University Hospital Ethics Committee and all patients signed an informed consent form.

### Serum IFX Assay

Serum IFX levels were measured by a capture ELISA as previously described [5]. Coating antibody (MoAb7 anti-TNF) and buffer (High Performance ELISA buffer) were supplied by Sanquin (Amsterdam, The Netherlands). Recombinant TNF was supplied by Preprotech and biotinylated anti-idiotype monoclonal antibody for detection was supplied by Progenika Biopharma (Derio, Spain). Peroxidase-Streptavidin and TMB were used for reaction developing, and optical density was read at 450 nm.

 The cut-off for positivity was 10ng/ml, established with a control group of 250 sera (150 from healthy donors and 100 from untreated RA patients). IFX serum levels were evaluated at baseline (W0) and at W2, W6, W14, W22 and W54 after initiation of IFX treatment. Serum samples from the 6 time points were available from 43 patients, missing a maximum of 2 samples /patient in the rest.

To determine whether the quantitative or qualitative data obtained by our capture ELISA could be extrapolated, we compared our results from University Hospital La Paz (UHLP) with two different commercial ELISAs; Pomonitor-IFX (Progenika Biopharma, Derio, Spain) and IFX ELISA-Compact produced by Sanquin (Amsterdam, The Netherlands). The Pearson´s correlation coefficients were 0.97 and 0.88, respectively (p<0.001 in each comparison), and no discrepancies in positive or negative results were found among all three assays (Kappa correlation index ULPH vs Sanquin =1; Kappa UHLP vs Promonitor=1) Fig. (**S1**).

 The agreement between drug levels of the three different assays was analyzed using an intra-class correlation coefficient (ICC); UHLP vs Sanquin (0.907; 95%IC 0.87-0.93; p<0.001) and UHLP vs Promonitor (0.64; 95%IC 0.39-0.78; p<0.001). The Bland-Altman plot Fig. (**S2**) gave an average of differences with a 95% limit of agreement, indicating that the two assay methods produce similar results.

### ATI Detection

ATI levels were assessed in sera from W0, W2, W6, W14 and W22 of treatment in 2 different ways:

An in-house 2-site (bridging) ELISA was used to detect uncomplexed (free) ATI as previously described [5]. The cut-off value for the presence of ATI was 50 arbitrary units (AU)/mL, established from the same serum control group as for IFX levels.

A commercial kit called IDKmonitor (Immundiagnostik, Bensheim, Germany) (A), and an acid-dissociation radioimmunoassay (ARIA, Sanquin) (B), were used to measure total (free and complexed) ATI. The (A) assay performed acid dissociation of the serum to acquire free ATI. Peroxidase conjugate-IFX and biotinylated-IFX were added to replace the unmarked therapeutic antibody and the marked IFX formed a complex with ATI that bound via biotin to a streptavidin-coated microtiter plate. Qualitative results higher than 10 AU/mL were considered positive. In the (B) assay, an acid dissociation was also performed to acquire free ATI. Samples were neutralized in a pH 7.6 buffer [20] with an excess of IFX F(ab´)_2_-biotin (to replace the unmarked therapeutic antibody). Antibodies were subsequently captured by Protein A Sepharose and radiolabelled streptavidin was used to detect specific ATI, analogue to the adalimumab ARIA [20].

Because of the retrospective nature of the study not all serum samples were available to be tested in all assays for ATI. Available samples were as follows: W0 (60 by brigding ELISA, 37 by ARIA and 59 by IDKmonitor); W2 (60 by brigding ELISA, 58 by ARIA and 60 by IDKmonitor); W6 (64 by brigding ELISA, 63 by ARIA and 63 by IDKmonitor); W14 (61 by brigding ELISA, 51 by ARIA and 58 by IDKmonitor); W22 (60 by brigding ELISA, 51 by ARIA and 57 by IDKmonitor).

### Statistical Analysis

Descriptive statistics were reported as mean and standard deviation (SD) or median and interquartile range (IQR) depending on normality. Patients were classified into two groups depending on the presence or absence of circulating serum IFX at W54, due to the ATI presence is detected in the most of patients during the first year of the treatment. In addition, we extended the follow-up time to W54 to include not only patients who develop primary inefficacy but also patients who develop secondary inefficacy associated with the development of ATI.

 Differences in clinical and biological characteristics between both groups were assessed using Pearson’s chi-square test or Fisher’s exact test for categorical variables and the Mann-Whitney U test or the Student t-test for continuous variables. Last observation carried forward (LOCF) was employed to impute data for patients who discontinued treatment before the end of the first year.

Serum-dependent receiver operating characteristic (ROC) curves at W2, W6, W14 and W22 were used to determine the ITL that best predicted the absence of free IFX at W54. The predictive cut-off was determined as the value for IFX levels that showed maximum sensitivity, maximum specificity and higher positive likelihood ratio at each studied time point. IFX treatment survival was studied using Kaplan-Meier curves and groups were compared using the log-rank test.

Univariable and multivariable logistic regression models were employed to investigate the association between early-stage ITL cut-off determined by ROC curves analysis and IFX at W54. Demographic, clinical and serological characteristics (including ITL cut-off at W2 and W6 as categorical variables) were included in the models as independent variables and the IFX levels as the dependent variable.

For all the analysis, GraphPad Prism 6 (San Diego, CA, USA) and SPSS 21.0 software were employed and p-values <0.05 were considered statistically significant.

## RESULTS

### 1 Patient Characteristics

Baseline characteristics for the 66 patients with RA starting IFX are summarized in Table **1**. Most patients were female (86%) and were positive for both rheumatoid factor (82%) and anti–cyclic citrullinated peptide antibody (82%). At inclusion time, all patients had active disease (DAS28: 5.5±1.3).

 Median disease duration prior to IFX treatment was 14 years (IQR: 9-18). Out of the 66 patients, 42 (64%) received concomitant treatment with methotrexate (MTX, mean dose 15 mg/week). Eight out of 66 (12%) patients discontinued the IFX treatment before the first year (5 due to lack of efficacy and 3 due to adverse events).

Patients were divided into 2 groups depending on the presence (ITLpos) or absence (ITLneg) of circulating serum IFX at W54 Table **1**. At W54, ITL were undetectable in 25 patients (38%), 23 of whom were ITLneg already since W22. ITLpos patients had lower baseline DAS28 scores compared to ITLneg patients (5.3±1.1 vs 5.8±1.2; p=0.06) and their mean (SD) IFX levels at W54 were 2.1 (1.8) µg/mL.

 Less ITLneg patients were treated with methotrexate (MTX) (11 (26%)) than ITLpos patients (32 (74%)); p≤0.01. ITLpos patients were older than ITLneg patients (59 years (51-69) vs 51 years (42-65)); p≤0.05. And the baseline C-reactive protein (CRP) was higher in ITLneg patients than in ITLpos patients (23±2 vs 15±17; p≤0.05).

### 2 Association Between Early-stage ITL and W54 IFX Status

The ITLneg patients at W54 had lower early-stage ITL than the ITLpos patients (W2: 20.0 ±12.7 µg/mL vs 29.7±14.5 µg/mL (p=0.015); W6: 4.2±5.9 µg/mL vs 15.7±11.1 µg/mL (p<0.0001); W14: 0.1±0.2 µg/mL vs 4.1±5.3 µg/mL (p<0.0001); and W22: 0.01±0.04 µg/mL vs 2.8±3.3 µg/mL (p<0.0001)) Fig. (**1A**).

 At all studied time points, the areas under the curve (AUC) of the ROC curves were statistically different from 0.5 Fig. (**1B**), enabling cut-off levels to distinguish between ITLpos and ITLneg patients at W54 (Table **2**).

 Out of the 25 ITLneg patients at W54, 12 (48%), 18 (72%) and 25 (100%) had ITL below the predictive cut-off at W2, W6 and W14, respectively. At W14, in 19 out of 25 patients (76%) ITL were undetectable. The W6 predictive cut-off (4.4 μg/mL) showed the best association with IFX absence at W54, with a sensitivity of 70% (95% confidence interval (CI): 45.7-88.1), a specificity of 95% (95% CI: 83.1-99.4) and a positive likelihood ratio of 14. Therefore, the W6 predictive cut-off was considered the reference value to predict clinical and serological outcomes for further analysis.

### 3 Analysis of Association between Early-stage ITL and other Confounder Factors with W54 IFX Status

In the univariable analysis, three factors were significantly associated with IFX absence at W54 Table **3A**: i) having an ITL below the cut-off at W2 (odds ratio (OR): 12.40; 95% CI: 3.48-44.15) or at W6 (OR: 44.33; 95% CI: 7.99-246.03), ii) non-use of MTX (OR: 4.20; 95% CI: 1.33-13.32) and iii) being older (OR: 1.04; 95% CI: 1.03-1.07).

In the multivariable logistic regression analysis, even after adjusting for possible confounders (age, gender and baseline DAS28), the ITL below the cut-off at W2 (OR: 15.85; 95% CI: 2.95-85.03; p=0.01) or at W6 (OR: 86.64; 95% CI: 6.58-1139.99) and also the non-use of MTX (OR: 12.26; 95% CI: 1.83-82.22) remained significantly associated with IFX absence at W54 Table **3B**.

## 4 Association between Early-stage ITL (W6) and Clinical Outcomes (W54)

Patients with ITL above the W6 predictive cut-off (4.4 µg/ml) had lower DAS28 scores at W54 than patients with ITL below the cut-off (3.68±1.26 vs 4.75±1.27; p=0.01) Fig. (**2A**). Most patients with low disease activity or remission by DAS28 at W54 had ITL above predictive cut-off at W6 (ITL above: 20 of 45 patients (44%) vs ITL below: 3 of 19 patients (16%); p=0.02) Fig. (**2B**).

 Likewise, most EULAR responders at W54 had ITL above the predictive cut-off at W6, namely 33 (73%) of 45 patients were EULAR responders with ITL above the cut-off vs 10 out of 19 EULAR responders (53%) with ITL below the cut-off; p=0.08 see Fig. (**S3**). When we analyzed the clinical outcome at W22 we found similar results.

 More patients with ITL at W6 above the predictive cut-off were in low disease activity or remission by DAS28 (3.75±1.09) than patients with ITL at W6 below the predictive cut-off (4.55±1.45); p=0.048 Fig. (**S2A**). We also found that 33 (73%) of 45 patients with ITL above the predictive cut-off at W6 were EULAR responders (good and moderate) while in patients with ITL below the cut-off at W6 t 9 (47%) of 19 were EULAR responders; p=0.075 see Fig. (**S2B**).

## 5 Association between IFX Survival and Early-stage Predictive Cut-off

Patients with ITL below the predictive cut-off at any of the studied time points dropped out of IFX treatment earlier (for patients with ITL above vs below: at W2, 4 years (1.1-3.6) vs 2 years (0.3-0.9), p=0.01; at W6, 5 years (1.6-5.0) vs 1.7 years (0.2-0.6), p=0.01; and at W14, 6.3 years (1.5-4.9) vs 2.3 years (1.1-3.8), p=0.03) see W6 data in Fig. (**2C**).

## 6 Association between Early-stage ATI Detection and W54 IFX Status

Most patients with ATI had ITL below the cut-off at W6 Table **4**. The IDKmonitor and ARIA drug-tolerant assays detected ATI production earlier than bridging ELISA, which only detected ATI if not complexed to IFX Table **4**. At baseline time, no patient had detectable ATI by any assayed methods. At W54, all the ITLneg patients were ATI-positive according to bridging ELISA.

## DISCUSSION

We have demonstrated that low ITL during induction phase are associated with IFX clearance and poor clinical outcomes after the first year of treatment in patients with RA. On top of ITL at W2 and especially at W6, non-use of MTX has also been observed to be predictive of IFX absence at W54. Furthermore, the early development of immunogenicity was found to be correlated with low ITL.

Some studies of patients with RA have reported an association between early IFX concentrations, drug maintenance throughout treatment and the detection of immunogenicity [12, 21]. Ducourau *et al.* [12], showed that low IFX concentrations from W2 to W14 were associated with increased ATI development and lower drug survival in patients with RA and patients with spondyloarthritis. Bendtzen *et al.* [21], reported that low IFX levels 6 weeks after starting therapy were predictive of ATI detection and the consequent loss of circulating IFX at 6 months of treatment in patients with RA. From our cohort, we could confirm that patients with low early-stage ITL were more prone to circulating drug loss after 1 year of treatment. Based on the results, this is the first time where a cut-off value of 4.4 μg/mL for ITL at W6 is defined as a predictor of efficacy and drug survival at W54 in RA patients.

**Fig. (S3):**
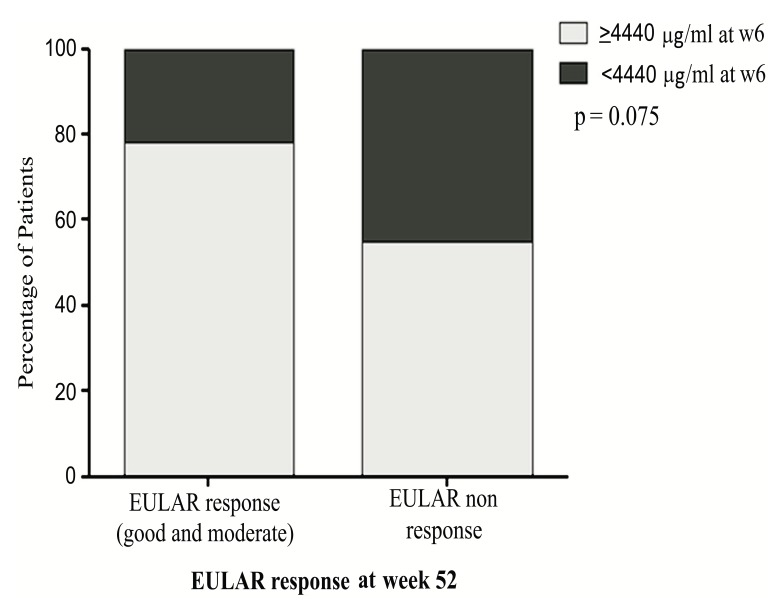
**EULAR response at W54.** Patients (%) with EULAR response (good and moderate) at W54, according to the week 6 ITL cut-off.

**Fig. (S4):**
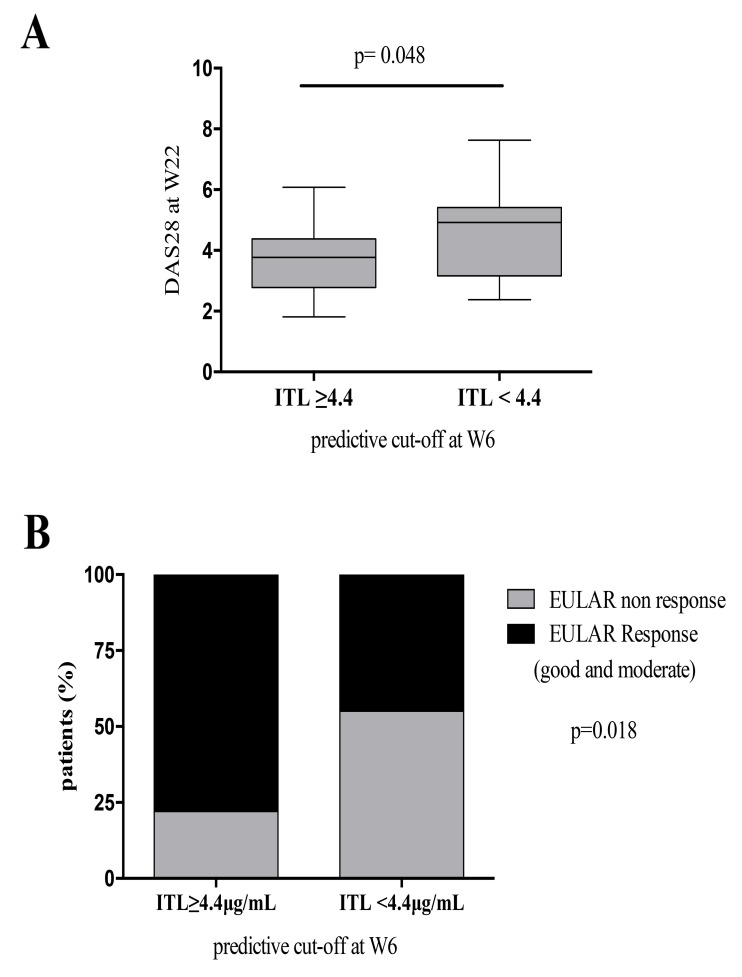
A) Disease Activity Score in 28 joints (DAS28) (median and interquartile ranges) after 22 weeks of IFX treatment for patients with rheumatoid arthritis (RA) with infliximab (Ifx) trough levels (ITL) above and below the week 6 predictive cut-off. B) Patients (%) with EULAR response (good and moderate) at W22, according to the week 6 ITL cut-off.

Although low ITL during treatment have been reported as being associated with therapy failure in several publications [5, 11, 22], there are only limited data available for the association between early-stage ITL and clinical response throughout treatment [11, 14, 23]. Mulleman *et al.* [11], described an association between IFX concentrations during the first months of treatment and clinical response. Kobayashi *et al.* [14], reported that on the basis of a controlled trial of 208 patients with UC, ITL at W2 above 21.3 µg/mL were significantly associated with both 14-week remission and 30-week mucosa healing. They also postulated that early-stage ITL could be used to predict treatment outcomes in UC patients. Van den Bemt *et al.* [23], showed from a cohort of 57 patients with RA that ITL at W6 together with disease activity scores optimized early detection of non-responders to IFX therapy. Other studies including patients with psoriasis and RA monitored adalimumab and etanercept levels at treatment initiation in which a significant positive association between serum drug levels and clinical response was observed [24, 25]. The clinical outcome analyzed at W22, was similar than at W54 (Fig. **S3**). However, in this work only the association of early ITL with the clinical outcome at W54 is considered,to include patients who develop secondary inefficacy associated with the development of ATI. In our study, we found that low ITL at W6 were associated with poor clinical outcomes at W54: i) Forty-eight percent of non-responder patients (according to EULAR criteria) at W54 had low serum ITL at the cut-off at W6; ii) Patients with low ITL at W6 had significantly higher disease activity (measured by DAS28) at W54; and iii) (Fig. **S4**). Patients with low early-stage ITL also had shorter drug survival rates. Therefore, our findings suggest a first cut-off value for ITL at induction phase that could be useful to predict not only IFX clearance but also poor clinical outcomes.

Several baseline demographic, clinical and serological factors have been described to influence on IFX levels, including body mass index [25]. From all the characteristics that we analyzed, only the age and the non-use of concomitant drugs were significantly associated with IFX clearance after the first year of treatment. However, in the multivariable analysis, only the non-use of MTX was found to be associated, which is consistent with published data. MTX appears to reduce immunogenicity and consequently helps to maintain high IFX levels [1, 9, 12, 21]. In this sense, Bendtzen *et al*. [21] found that ATI-positive RA patients receiving MTX had lower antibody levels than patients not receiving MTX. By these findings, we suggest that MTX use should be considered by the clinicians when possible, due to its beneficial effect in rheumatoid arthritis patients under biological therapy.

Previous studies have shown that low ITL are associated with the development of immunogenicity [5, 22, 26]. However, the detection of antidrug antibodies varies widely depending on the assay used, mainly because of drug interference [13, 20]. For bridging ELISA, ATI detection can be expected to be small in the IFX induction phase because antibody presence is underestimated in the presence of high drug concentration. Therefore, to determine whether low early-stage ITL were due to early ATI production, in this study ARIA and IDKmonitor drug-tolerant assays were employed. Both methods detected complexed ATI as early as at W2. Most patients positive for early-stage complexed ATI had ITL below the predictive cut-off at W6. Recent interest has focused on detecting free as well as complexed ATI. The authors of a study in RA patients using a drug-tolerant assay to analyze anti-adalimumab production concluded that many early antibodies may be transient and therefore may not influence the clinical efficacy of the treatment [27, 28]. However, our study supports the idea that complexed ATI detection in the initial treatment weeks could be useful to identify an early development of immunogenicity.

Our study has several limitations. Among them, its retrospective design and the relative small sample size are the most relevant to consider when interpreting the results. However, despite the limited number of included patients, serum samples were available at many different time points (W2, W6, W14, W22 and W54). Our results were obtained using in-house ELISA [5] to measure IFX levels, however, the correlation with two commercially available ELISA kits to measure IFX makes our results extrapolable. Indeed, in real practice, cut-off values can never be taken as an exact value but as an approach indicating a magnitude above or below. Each laboratory should establish its own cut-off values depending on the method used to quantify IFX serum levels, but always considering the relevance of the predictive value of the levels at early weeks (W2 and W6) on the treatment outcome.

## CONCLUSION

In conclusion, to our knowledge, this is the first study with RA patients that establishes an association between early-stage ITL (at W2 and W6) and IFX loss, early treatment dropout and clinical outcomes after the first year of treatment. Furthermore, it is also suggested that ATI development is the main reason for low early-stage circulating IFX levels. Finally, a cut-off value for ITL at W6 is also proposed. This cut-off value could provide clinicians a useful tool to predict treatment efficacy in patients with RA treated with IFX.

Author contributions: TJ, CP-R and DP-S were involved in the study design, the patients´ selection and data collection. TJ, DP-S, CP-R and AM-F wrote the article. AB, VN-C, KB and TR reviewed the article. CP-R, AV, M-GB, LN and DP and were directly involved in patient management. AM-F, EO, CD, AV and KB performed the serum assays. All authors read and approved the final manuscript.

## Figures and Tables

**Fig. (1):**
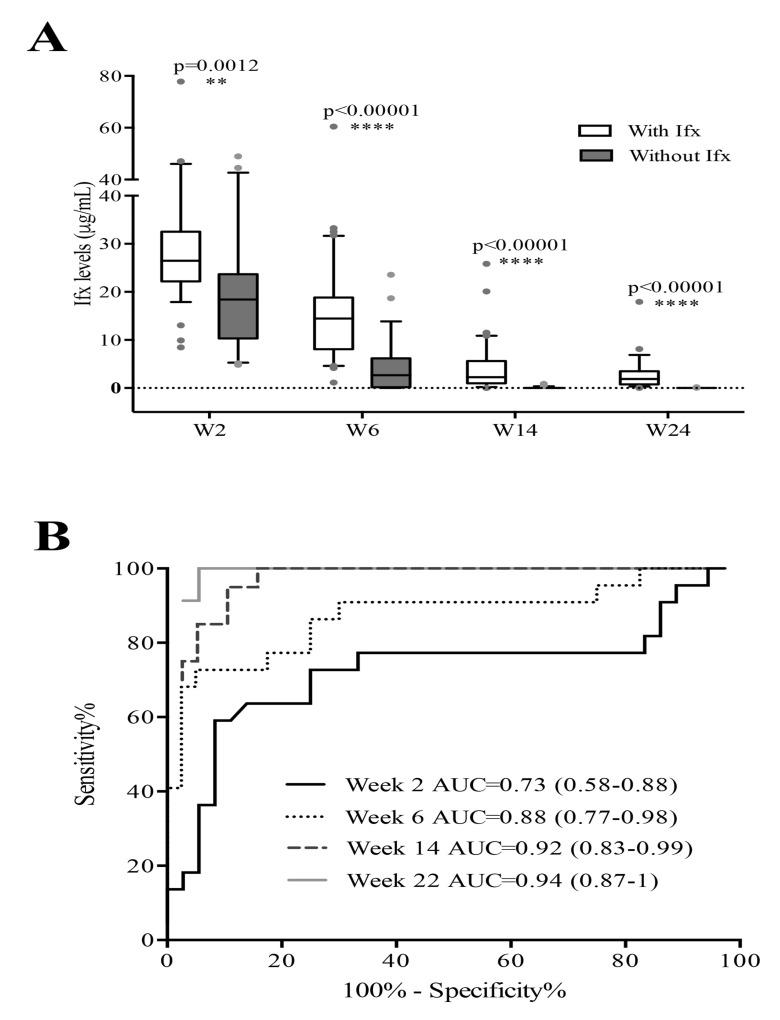
A) Infliximab (Ifx) trough levels (ITL) in patients with rheumatoid arthritis at early treatment stages, according to week (W) 54 Ifx status. Results are shown as medians (solid lines within boxes), interquartile ranges (upper and lower box boundaries) and maximums and minimums. Black dots indicate outliers. B) Receiver operating characteristic curves for ITL at W2, W6, W14 and W22 for prediction of Ifx absence at W54. AUC, Area Under the Curve.

**Fig. (2):**
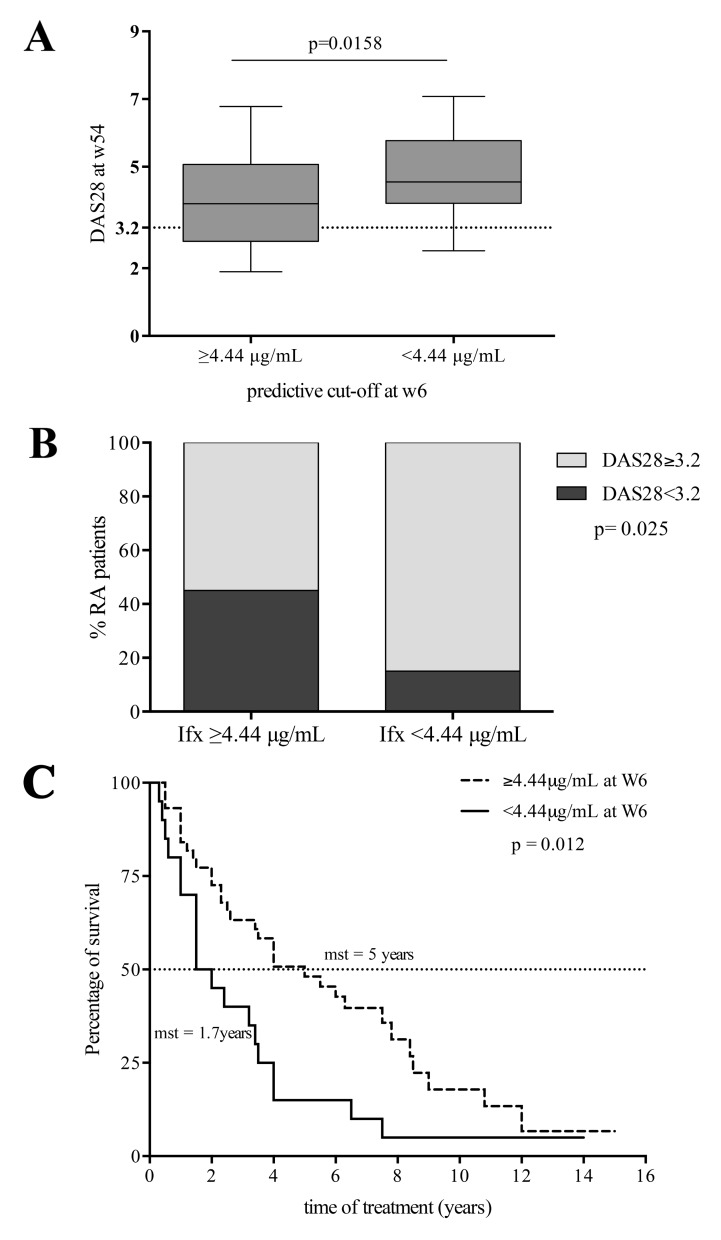
A) Disease Activity Score in 28 joints (DAS28) (median and interquartile ranges) for patients with rheumatoid arthritis (RA) with infliximab (Ifx) trough levels (ITL) above and below the week 6 predictive cut-off. B) Patients (%) with low disease activity or remission (DAS28 < 3.2) at W54, according to the week 6 ITL cut-off. C) Kaplan-Meier curves for Ifx treatment survival, according to the week 6 predictive cut-off. Differences between groups were evaluated by the log- rank test. Mean survival time (mst).

**Fig. (S1):**
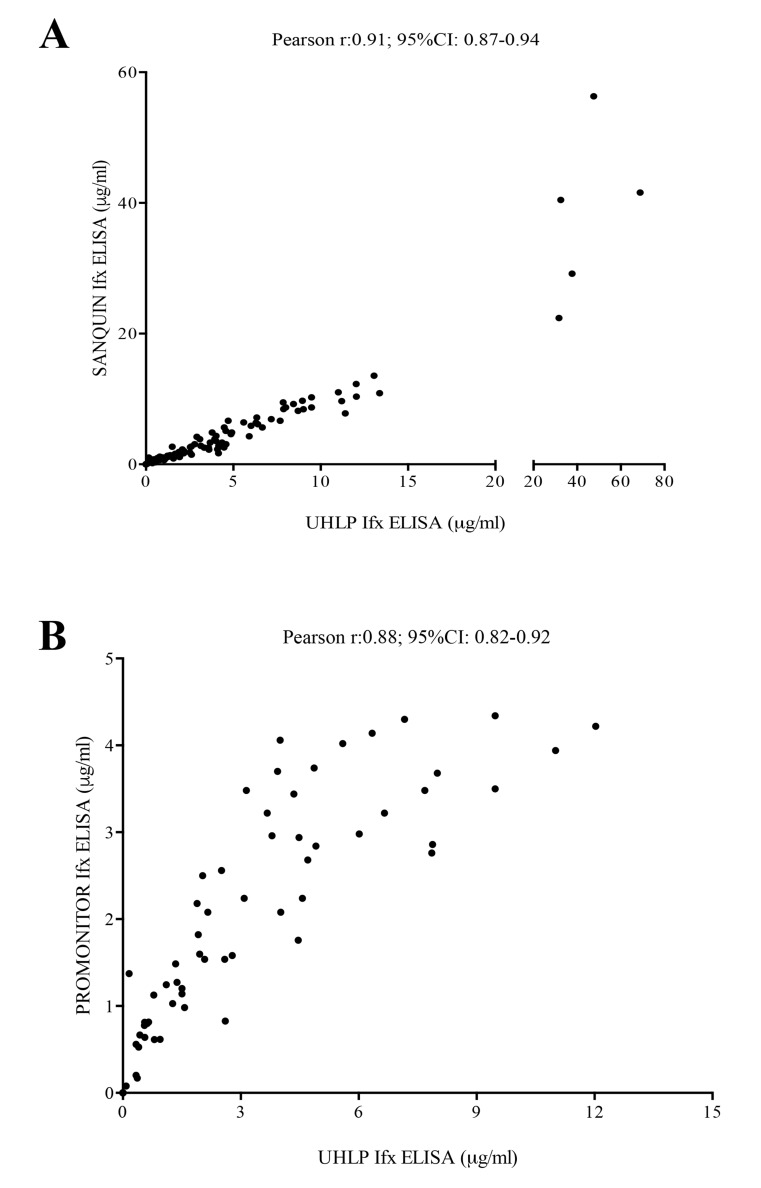
**Correlation between UHLP capture ELISA and two different ELISAs (PROMONITOR and SANQUIN). A)** Infliximab (IFX) trough levels (ITL) in patients with rheumatoid arthritis (n=124) measured by UHLP capture ELISA and SANQUIN ELISA. Results are shown correlations between both assays. **B)** Infliximab (IFX) trough levels (ITL) in patients with rheumatoid arthritis (n=77) measured by UHLP capture ELISA and PROMONITOR ELISA. Results are shown correlations between both assays.

**Fig. (S2):**
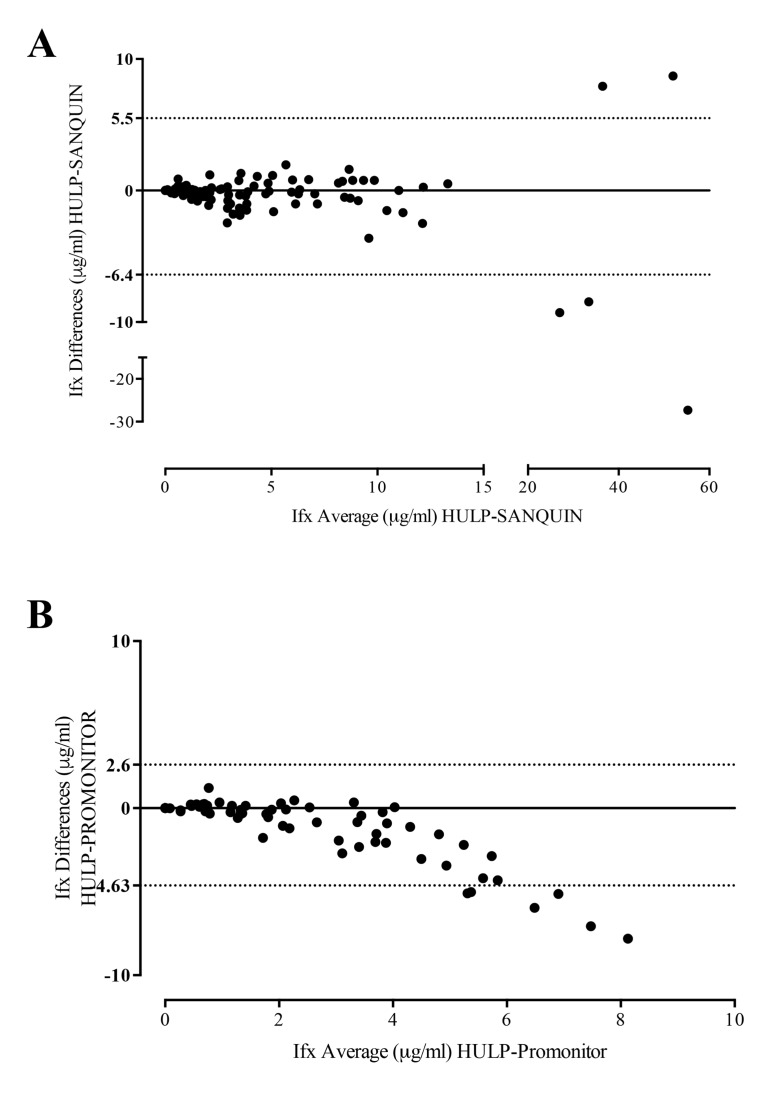
**Comparison of IFX methods by Bland-Altman analysis. A)** Bland-Altman analysis between UHLP and SANQUIN, the differences between the two measurements (Y-axis in µg/ml) is plotted against the average of each pair of measurements (X-axis in µg/ml), the 95% limits of agreement are represented by dot line. **B)** Bland-Altman analysis between UHLP and Promonitor, the differences between the two measurements (Y-axis in µg/ml) is plotted against the average of each pair of measurements (X-axis in µg/ml), the 95% limits of agreement are represented by dot line.

**Table 1:** Demographic characteristics of 66 patients with rheumatoid arthritis (RA).

Characteristics	Patients (n=66)	ITLpos at week 54 (n=41)	ITLneg at week 54 (n=25)	p
Age, years*	56 (47-68)	59	51 (42-65)	<0.05
Body mass index*	24 (22-27)	24 (22-27)	23(20-28)	0.2
Female**	57 (86%)	35 (85%)	22 (88%)	1
Disease duration, years*	14 (9-18)	16 (10-19)	12 (5-16)	0.1
Rheumatoid factor**	54 (82%)	33(80%)	22(96.6%)	0.3
ACPA**	54 (82%)	32(82%)	22(88%)	0.2
DAS28 at baseline***	5.5 (1.3)	5.3 (1.1)	5.8 (1.4)	0.06
CRP at baseline***	18 (19)	15 (17)	23 (20)	0.05
Concomitant treatment:				
Methotrexate**	43 (65%)	32 (78%)	11 (44%)	<0.01
Methotrexate dose, mg/week*	15.6 (5.5)	12.2 (8.5)	8.8 (7.4)	0.1
Others DMARDs **	16 (24%)	7 (17%)	9 (36%)	0.6
Prednisone**	43 (65%)	31 (75%)	12 (48%)	<0.05
Monotherapy **	8 (12%)	5 (12%)	3(15%)	1

*Median (interquartile range); ** n (%); *** mean (standard deviation)

Body mass index(kg/m^2^); ACPA, anti-citrullinated protein antibodies(IU/ml); CRP, C-reactive protein(mg/L); DAS28, Disease Activity Score in 28 joints; DMARD, disease-modifying anti-rheumatic drug; IL-6

**Table 2:** Early-stage treatment Infliximab level cut-offs with sensitivity, specificity and positive likelihood ratio (LR+) values.

Week	Cut-off	Sensitivity (95% CI)	Specificity (95% CI)	LR+
2	21.2 μg/mL	67% (44-84)	86% (70-95)	4.8
6	4.4 μg/mL	70% (45-88)	95% (83-99)	14
14	0.4 μg/mL	83% (35-99)	89% (75-97)	7.9
22	0.2 μg/mL	100% (15-100)	94% (81-99)	18

CI, confidence interval; LR, Likelihood Ratio.

**Table:** A. Univariable analyses for clinical baseline factors and early-stage infliximab (IFX) trough levels (ITL) for ITL absence at week (W) 54.

Factors	OR	95%CI
At baseline		
Female sex	1.59	0.29-8.69
Age	1.04	1.03-1.07
Rheumatoid factor	0.54	0.13-2.18
ACPA	0.21	0.24-1.81
Body mass index	1.05	0.93-1.18
DAS28	0.70	0.46-1.05
MTX non-use	4.20	1.33-13.32
CRP levels	0.98	0.95-1.05
Levels below cut-off at W2 (21.2 μg/mL)	12.40	3.48-44.15
Levels below cut-off at W6 (4.4 μg/mL)	44.33	7.99-246.03

**Table:** B. Multivariable logistic regression analysis for IFX absence at W54 including ITL at W2 (Model I) and at W6 (Model II) as possible predictors.

Factors	OR	95%CI
Model I		
Female sex	1.09	0.09-13.18
Age	1.09	1.03-1.17
DAS28	0.64	0.36-1.14
MTX non-use	12.26	1.83-82.22
Levels below cut-off at W2 (21.2 μg/mL)	15.85	2.95-85.03
Model II		
Female sex	0.65	0.05-7.97
Age	1.05	0.98-1.12
DAS28	0.64	0.31-1.30
MTX non-use	6.91	1.04-45.84
Levels below cut-off at W6 (4.4 μg/mL)	86.64	6.58-1139.99

ACPA, anti-cyclic citrullinated peptide antibodies; CRP, C-reactive protein; CI, confidence interval; DAS28, Disease Activity Score in 28 joints; MTX, methotrexate; OR, odds ratio; TNF-α, tumour necrosis factor α; ITL, Infliximab trough levels.

**Table 4:** ATI Frequency Assayed by three Different Methods for all Studied Time Points.

	[IFX] μg/mL at W6	bridging ELISA	IDKmonitor	ARIA
W2	IFX ≥4.4 n=44	0	0	0
	IFX <4.4 n=20	0	1 (5%)	1 (5%)
	p	1	0.33	0.33
W6	IFX ≥4.4 n=44	0	1 (2.3%)	2 (4.5%)
	IFX <4.4 n=20	4 (20%)	7 (35%)	10 (50%)
	p	0.007	0.002	0.001
W14	IFX ≥4.4 n=44	2 (4.5%)	7 (16%)	12 (27.3%)
	IFX <4.4 n=20	11 (55%)	16 (80%)	16 (80%)
	p	<0.0001	<0.0001	0.0006
W22	IFX ≥4.4 n=44	5 (11.4%)	20 (45.5%)	25 (56.8%)
	IFX <4.4 n=20	15 (75%)	18 (90%)	18 (90%)
	p	<0.0001	<0.0001	0.01
W54	IFX ≥4.4 n=44	5 (11.4%)	22(50%)	27(61.3%)
	IFX <4.4 n=20	15 (75%)	18 (90%)	18 (90%)
	p	<0.0001	0.002	0.02

ARIA, acid-dissociation radioimmunoassay (Sanquin, Amsterdam, The Netherlands); IDKmonitor, commercial kit (Immundiagnostik, Bensheim, Germany); IFX, infliximab; W, week.

## References

[1] Maini R.N., Breedveld F.C., Kalden J.R., Smolen J.S., Davis D., Macfarlane J.D. (1998). Therapeutic efficacy of multiple intravenous infusions of anti-tumour necrosis factor a monoclonal antibody combined with low-dose weekly methotrexate in rheumatoid arthritis.. Arthritis Rheum..

[2] Braun J., Brandt J., Listing J., Zink A., Alten R., Burmester G. (2003). Long-term efficacy and safety of infliximab in the treatment of ankylosing spondylitis: an open, observational, extension study of a three-month, randomized, placebo-controlled trial.. Arthritis Rheum..

[3] Van Der Heijde D., Braun J., Deodhar A., Dijkmans B., Geusens P., Sieper J., Williamson P., Xu W., Visvanathan S., Baker D., Goldstein N. (2008). Efficacy and safety of infliximab in patients with ankylosing spondylitis over a two-year period.. Arthritis Care & Research..

[4] Hanauer S.B., Feagan B.G., Lichtenstein G.R., Mayer L.F., Schreiber S., Colombel J.F. (2002). Maintenance infliximab for Crohn's disease: the ACCENT I randomised trial.. Lancet.

[5] Pascual-Salcedo D., Plasencia C., Ramiro S., Nuño L., Bonilla G., Nagore D. (2011). Influence of immunogenicity on the efficacy of long-term treatment with infliximab in rheumatoid arthritis.. Rheumatology.

[6] Mulleman D., Méric J-C., Paintaud G., Ducourau E., Beuzelin C-M., Valat J-P. (2009). Infliximab concentration monitoring improves the control of disease activity in rheumatoid arthritis.. Arthritis Res. Ther..

[7] Bartelds G.M., Krieckaert C.L., Nurmohamed M.T., van Schouwenburg P.A., Lems W.F., Twisk J.W. (2011). Development of antidrug antibodies against adalimumab and association with disease activity and treatment failure during long-term follow-up.. JAMA.

[8] Wolbink G.J., Vis M., Lems W.F., Voskuyl A.E., de Groot E., Nurmohamed M.T. (2006). Development of anti-infliximab antibodies and relationship to clinical response in patients with rheumatoid arthritis.. Arthritis Rheum..

[9] Plasencia C., Pascual-Salcedo D.F., Nuno L.F., Bonilla G.F., Villalba A.F., Peiteado D.F. (2012). Influence of immunogenicity on the efficacy of longterm treatment of spondyloarthritis with infliximab.. Ann. Rheum. Dis..

[10] St Clair E.W., Wagner L.C., Fasanmade A.A., Wang B., Schaible T., Kavanaugh A. (2002). The relationship of serum infliximab concentrations to clinical improvement in rheumatoid arthritis.. Arthritis Rheum..

[11] Mulleman DF, Chu Miow Lin DF, Ducourau E (2010). Trough infliximab concentrations predict efficacy and sustained control of disease activity in rheumatoid arthritis.. Ther. Drug Monit..

[12] Ducourau E., Mulleman D., Paintaud G. (2011). Antibodies toward infliximab are associated with low infliximab concentration at treatment initiation and poor infliximab maintenance in rheumatic diseases.. Arthritis Res. Ther..

[13] Hart MH (2011). Differential effect of drug interference in immunogenicity assays.. J. Immunol. Methods.

[14] Kobayashi T., Suzuki Y., Motoya S., Hirai F., Ogata H., Ito H., Sato N., Ozaki K., Watanabe M., Hibi T. (2016). First trough level of infliximab at week 2 predicts future outcomes of induction therapy in ulcerative colitis—results from a multicenter prospective randomized controlled trial and its post hoc analysis.. Journal of gastroenterology..

[15] Ternant D., Ducourau E., Perdriger A., Corondan A., Le Goff B., Devauchelle‐Pensec V., Solau‐Gervais E., Watier H., Paintaud G., Mulleman D. (2014). Relationship between inflammation and infliximab pharmacokinetics in rheumatoid arthritis.. British journal of clinical pharmacology..

[16] Ternant D., Aubourg A., Magdelaine-Beuzelin C., Degenne D., Watier H., Picon L. (2008). Infliximab pharmacokinetics in inflammatory bowel disease patients.. Ther. Drug Monit..

[17] Arnett F.C., Edworthy S.M., Bloch D.A. (1988). The American Rheumatism Association 1987 revised criteria for the classification of rheumatoid arthritis.. Arthritis Rheum..

[18] Prevoo M.L., van 't Hof M.A., Kuper H.H., van Leeuwen M.A., van de Putte L.B., van Riel P.L. (1995). Modified disease activity scores that include twenty-eight-joint counts. Development and validation in a prospective longitudinal study of patients with rheumatoid arthritis.. Arthritis Rheum..

[19] van Gestel A., Prevoo M., van ´t Hof M., van Rijswijk M., van de Putte L., van Riel P. (1996). Development and validation of the European League Against Rheumatism response criteria for rheumatoid arthritis. Comparison with the preliminary American College of Rheumatology and the World Health Organization/International League Against Rheumatism Criteria.. Arthritis Rheum..

[20] Bloem K (2015). Systematic comparison of drug-tolerant assays for anti-drug antibodies in a cohort of adalimumab-treated rheumatoid arthritis patients.. J. Immunol. Methods.

[21] Bendtzen K., Geborek P., Svenson M., Larsson L., Kapetanovic M.C., Saxne T. (2006). Individualized monitoring of drug bioavailability and immunogenicity in rheumatoid arthritis patients treated with the tumor necrosis factor alpha inhibitor infliximab.. Arthritis Rheum..

[22] van der Maas A., van den Bemt B.J., Wolbink G., van den Hoogen F.H., van Riel P.L., den Broeder A.A. (2012). Low infliximab serum trough levels and anti-infliximab antibodies are prevalent in rheumatoid arthritis patients treated with infliximab in daily clinical practice: results of an observational cohort study.. BMC Musculoskelet. Disord..

[23] van den Bemt B.J., den Broeder A.A., Wolbink G.J. (2013). The combined use of disease activity and infliximab serum trough concentrations for early prediction of (non-)response to infliximab in rheumatoid arthritis.. Br. J. Clin. Pharmacol..

[24] Jamnitski AF., Krieckaert CL., Nurmohamed MT., Hart MH., Dijkmans BA., Aarden L., Voskuyl AE., Wolbink GJ. (2011). Patients non-responding to etanercept obtain lower etanercept concentrations compared with responding patients.. Annals of the rheumatic diseases..

[25] Mahil S.K., Arkir Z., Richards G., Lewis C.M., Barker J.N., Smith C.H. (2013). Predicting treatment response in psoriasis using serum levels of adalimumab and etanercept: a single-centre, cohort study.. Br. J. Dermatol..

[26] Ben-Horin S., Yavzori M., Katz L. (2011). The immunogenic part of infliximab is the F(ab')2, but measuring antibodies to the intact infliximab molecule is more clinically useful.. Gut.

[27] van Schouwenburg P.A., Bartelds G.M., Hart M.H., Aarden L., Wolbink G.J., Wouters D. (2010). A novel method for the detection of antibodies to adalimumab in the presence of drug reveals “hidden” immunogenicity in rheumatoid arthritis patients.. J. Immunol. Methods.

[28] van Schouwenburg P.A., Krieckaert C.L., Rispens T., Aarden L., Wolbink G.J., Wouters D. (2013). Long-term measurement of anti-adalimumab using pH-shift-anti-idiotype antigen binding test shows predictive value and transient antibody formation.. Ann. Rheum. Dis..

